# The Rice Eukaryotic Translation Initiation Factor 3 Subunit f (OseIF3f) Is Involved in Microgametogenesis

**DOI:** 10.3389/fpls.2016.00532

**Published:** 2016-04-26

**Authors:** Qi Li, Zhuyun Deng, Chunyan Gong, Tai Wang

**Affiliations:** ^1^Key Laboratory of Plant Molecular Physiology, Institute of Botany, Chinese Academy of SciencesBeijing, China; ^2^University of Chinese Academy of SciencesBeijing, China

**Keywords:** pollen development, microgametogenesis, translation initiation, eIF3f, rice

## Abstract

Microgametogenesis is the post-meiotic pollen developmental phase when unicellular microspores develop into mature tricellular pollen. In rice, microgametogenesis can influence grain yields to a great degree because pollen abortion occurs more easily during microgametogenesis than during other stages of pollen development. However, our knowledge of the genes involved in microgametogenesis in rice remains limited. Due to the dependence of pollen development on the regulatory mechanisms of protein expression, we identified the encoding gene of the eukaryotic translation initiation factor 3, subunit f in *Oryza sativa* (OseIF3f). Immunoprecipitation combined with mass spectrometry confirmed that OseIF3f was a subunit of rice eIF3, which consisted of at least 12 subunits including eIF3a, eIF3b, eIF3c, eIF3d, eIF3e, eIF3f, eIF3g, eIF3h, eIF3i, eIF3k, eIF3l, and eIF3m. *OseIF3f* showed high mRNA levels in immature florets and is highly abundant in developing anthers. Subcellular localization analysis showed that OseIF3f was localized to the cytosol and the endoplasmic reticulum in rice root cells. We further analyzed the biological function of OseIF3f using the double-stranded RNA-mediated interference (RNAi) approach. The OseIF3f-RNAi lines grew normally at the vegetative stage but displayed a large reduction in seed production and pollen viability, which is associated with the down-regulation of *OseIF3f*. Further cytological observations of pollen development revealed that the OseIF3f-RNAi lines showed no obvious abnormalities at the male meiotic stage and the unicellular microspore stage. However, compared to the wild-type, OseIF3f-RNAi lines contained a higher percentage of arrested unicellular pollen at the bicellular stage and a higher percentage of arrested unicellular and bicellular pollen, and aborted pollen at the tricellular stage. These results indicate that *OseIF3f* plays a role in microgametogenesis.

## Introduction

Rice (*Oryza sativa* L.) is a staple food for half of the world’s population and has become a model monocot for research in plant biology. Pollen development is an important biological process in the sexual propagation of rice. Whether normal pollen grains can be formed through stage-sequential developmental processes has a direct effect on the number of filled grains per panicle and thus influences grain production. Furthermore, male sterile materials with pollen abortion greatly facilitate the maintenance and production of hybrid rice showing good agronomic traits. Therefore, understanding the mechanisms of pollen development is crucial to rice culture. During pollen development, two different and successive stages, namely microsporogenesis and microgametogenesis, result in the production of mature pollen grains. Microsporogenesis begins with the formation of pollen mother cells, which divide by meiosis to produce unicellular microspores. Afterwards, microspores undergo two rounds of mitosis during microgametogenesis. At pollen mitosis I, the unicellular microspore divides asymmetrically to generate bicellular pollen with a large vegetative cell and a small generative cell. The generative cell subsequently undergoes pollen mitosis II to produce two sperm cells enclosed within the vegetative cell, thus leading to the formation of mature tricellular pollen. Defects in any of these developmental events can possibly cause pollen abortion. Cytological study of male sterility has demonstrated that pollen abortion in rice tends to be associated with developmental defects occurring during microgametogenesis (Laser and Lersten, 1972). Transcriptomic analysis has identified a large number of genes expressed preferentially during microgametogenesis in rice (Wei et al., 2010; Wu et al., 2014); however, a limited number of genes have been functionally characterized and have been shown to function in microgametogenesis. Recent studies show that *OsRAD21-3*, *OsGEN-L* and *ZEP1*, encoding a rice ortholog of the yeast sister chromatid cohesion complex subunit Rad21, a rice member of the RAD2/XPG endonuclease family, and the central element of the rice synaptonemal complex, respectively, are involved in pollen mitosis (Moritoh et al., 2005; Tao et al., 2007; Wang et al., 2010). Genes required for pollen wall formation, such as *OsGT1* and *CAP1*, also play important roles in microgametogenesis (Moon et al., 2013; Ueda et al., 2013). In addition, *Osnop*, *RIP1*, and *RA68* are shown to be necessary for post-meiotic pollen formation, although their exact cellular and molecular functions are unclear (Jiang et al., 2005; Han et al., 2006; Li et al., 2010). These results indicate that microgametogenesis involves a series of coordinated cellular processes including pollen mitosis, pollen wall formation and various other events, which depend on precise and reliable translation of related genes.

Translation in eukaryotes is primarily regulated during the initiation phase, in which at least 12 eukaryotic initiation factors (eIFs) are involved (Jackson et al., 2010; Hinnebusch and Lorsch, 2012). The eIF3 complex is the largest initiation factor and serves as a scaffold in translation initiation. In *Arabidopsis* and wheat, eIF3 consists of 11 subunits (a, b, c, d, e, f, g, h, i, k, and l; Burks et al., 2001; Marchione et al., 2013). It is still unclear whether rice eIF3 is also constituted by these subunits and whether individual subunits of eIF3 are required for microgametogenesis in rice. In this study, we confirmed that OseIF3f can form a complex with other 11 subunits of eIF3, which include a, b, c, d, e, g, h, i, k, l, and m. Using the double-stranded RNA-mediated interference (RNAi) approach, we revealed that OseIF3f may have a role in microgametogenesis.

## Materials and Methods

### Plant Materials

The wild-type (WT) plant was rice cultivar Zhonghua 10 (*O. sativa* L. ssp. *japonica*). Roots, leaves, shoots and panicles were collected as described previously (Zhang et al., 2006). The developing florets at different stages were grouped according to the length of florets and panicles as described previously (Tao et al., 2007).

### Plasmid Construction and Plant Transformation

To construct the overexpression vector pHA-OseIF3f, DNA fragments encoding the N-terminal hemagglutinin (HA)-tagged OseIF3f protein was amplified with the primer P1F (the nucleotide sequences of all the primers used in this study are listed in **Supplemental Table S1**) and P1R, and then cloned into binary vector pUN1301 (Wang et al., 2004).

The *OseIF3f* promoter::beta-glucuronidase (GUS) plasmid was constructed by inserting a 1980-bp genomic DNA fragment upstream of *OseIF3f* open reading frame (ORF), which was amplified with P2F and P2R, into the binary vector pCAMBIA1391Xb (CAMBIA companies).

RNA-mediated interference tool vector pWTC615 is modified from pWTC605 (Zhang et al., 2006) by substituting an ubiquitin promoter (Wang et al., 2004) for the CaMV 35S promoter. The sense and antisense cDNA fragments of *OseIF3f* (566–855 bp) were amplified by using the primer P3F and P3R, and P4F and P4R, respectively, and then inserted into pWTC615 to generate the RNAi vector p6OseIF3fi. For construction of the second RNAi vector p3OseIF3fi, the 125–668 bp cDNA fragment of *OseIF3f* was amplified using P5F and P5R. The amplified cDNA fragment was double-digested with *Kpn*I/*BamH*I and *Spe*I/*Sac*I, respectively, and then inserted into the binary vector pTCK303 (Wang et al., 2004) in the sense and antisense direction, respectively.

The resulting binary plasmids mentioned above were, respectively, transformed into an *Agrobacterium tumefaciens* strain EHA105, which were used to transform rice embryonic calli and obtain regenerated plants (transgenic lines in T0 generation) according to the method described (Hiei et al., 1994). R1 generation plants were generated from axillary buds of their T0 plants after cutting leaves and stems off the mature T0 plants. Seeds obtained from the T0 plants were selected on 1/2 MS medium containing 25 mg/l hygromycin B (Roche) and the surviving T1 seedlings were grown in soil for further analysis.

### Immunoprecipitation and Protein Identification by Mass Spectrometry

Rice developing panicles from the transgenic plants overexpressing the HA-tagged OseIF3f were ground into fine powders in liquid nitrogen and mixed well with a cold lysis buffer [50 mM Tris-Cl, pH7.5, 150 mM NaCl, 10 mM MgCl_2_, 0.15% NP40, 1 mM PMSF, and 1× complete protease inhibitor (Roche)]. After adding Benzonase (Novagen), the lysates were incubated for 1 h at 4°C on a rocker and centrifuged twice at 20000 *g* for 15 min at 4°C. The supernatants containing the extracted proteins were pre-cleared with freshly prepared mouse IgG-coated Dynabeads Protein A (life technologies) for 4 h at 4°C, followed by immunoprecipitation with the freshly prepared monoclonal HA antibody-coated Dynabeads Protein A for 4 h at 4°C on a rotating rocker. The beads were washed thoroughly with the cold lysis buffer. Immunoprecipitates were eluted from the beads by boiling in 2× SDS sample buffer and resolved on a 12% SDS-PAGE gel. The immunoprecipitation experiments were repeated three times in parallel with a control IgG immunoprecipitation. Except for the two IgG bands (heavy and light chains), all the protein bands of the HA-OseIF3f and IgG immunoprecipitates were excised and digested with trypsin as described previously (Shevchenko et al., 2006) before identification by mass spectrometry.

Peptides were extracted from the gel matrix and subjected to liquid chromatography–tandem mass spectrometry (LC–MS/MS). The extracted peptide samples were separated by high-performance liquid chromatography (HPLC) on an Eksigent nano-LC Ultra (AB SCIEX). The samples was desalted on a 100 μm × 20 mm trap column and eluted on an analytical 75 μm × 150 mm column. Both columns were filled with MAGIC C18AQ 5 μm 200 Å phase (Michrom Bioresources). A linear gradient system that consisted of 0.1% formic acid in water (mobile phase A) and 0.1% formic acid in acetonitrile (mobile phase B) flowing from 5 to 30% mobile phase B for 30 min at a flow rate of 300 nL/min was employed for separation of peptides. The MS analysis was performed on a TripleTOF 5600+ system (AB SCIEX) in information dependent mode. MS spectra were acquired across the mass range of 350–1500 *m*/*z* in high resolution mode (>30000) using 250 ms accumulation time per spectrum. A maximum of 40 precursors per cycle were chosen for fragmentation from each MS spectrum with 50 ms minimum accumulation time for each precursor and dynamic exclusion for 18 s. Tandem mass spectra were recorded in high sensitivity mode (resolution >15000) with rolling collision energy on.

ProteinPilot (Version 4.5.0.0, AB SCIEX) was used for sample profiles with the Paragon method to search against NCBInr_20150104 database (54183042 sequences). Search parameters were set as follows: sample type set to identification, cysteine alkylation set to iodoacetamide, trypsin digestion, instrument set to TripleTOF 5600, special factors set to none, species *O. sativa*, and a thorough ID search effort. The protein confidence threshold cutoff was 2 (unused ProtScore), with at least two peptides with 95% confidence. The false discovery rate (FDR) <1% was set for protein identification.

### Real-time Quantitative Reverse Transcription PCR

Total RNAs were extracted using the Trizol Reagent (Invitrogen). First-stand cDNA was synthesized with SuperScript III Reverse Transcriptase (Invitrogen) by using the extracted total RNAs. First-stand cDNA was used to perform real-time quantitative reverse transcription PCR (real-time qRT-PCR) with primer pairs P6F and P6R for analysis of *OseIF3f* mRNA levels in different tissues and in p6OseIF3fi RNAi lines, P7F and P7R for analysis of *OseIF3f* mRNA levels in p3Oseif3fi RNAi lines, P8F and P8R for the internal control 18s rRNA, and P9F and P9R for the internal control tubulin (NCBI number: AK07252; Jain et al., 2006). Each real-time qRT-PCR amplification was performed in triplicate using Power SYBR Green PCR Master mix (life technologies) on a Step One Plus real-time PCR system (Life Technologies). The 2^-ΔΔC_t_^ method was used to calculate relative quantification of mRNA expression (Livak and Schmittgen, 2001).

### Subcellular Localization Analysis

To analyze subcellular localization with transiently expressed GFP-OseIF3f fusion proteins, the full ORF of *OseIF3f* was amplified using the primer P10F and P10R, then digested and cloned into pA7 (Sohlenkamp et al., 2002) to generate the CaMV 35S::GFP-OseIF3f transient expression vector. Afterwards, the purified CaMV 35S::GFP-OseIF3f vector and the ER-dsRed2 plasmid were bombarded into onion epidermis cells by using a biolistic PDS-1000/He particle delivery system (Bio-Rad). After incubating the onion cells in dark at 25°C for about 48 hours, fluorescent signals were detected under an Axio Imager A1 fluorescent microscope (Zeiss) equipped with an AxioCam MRc5 digital camera (Zeiss).

For immunolocalization of OseIF3f, roots from the transgenic plants overexpressing the HA-tagged OseIF3f protein were fixed, digested, squashed and hybridized with the anti-HA antibody and then with Tetramethylrhodamine (TRITC)-conjugated goat anti-mouse IgG antibodies using the described method (Tao et al., 2007). The hybridized root cells were stained with the ER-specific dye dihexyloxacarbocyanine iodide (DiOC6) before observation under the Zeiss Axio Imager A1 fluorescent microscope.

### Antibody Production and Western Blotting

The synthesized 14 residues (32–45 amino acids) of OseIF3f were crosslinked with keyhole limpet hemocyanin and used to immunize rabbits for generating the anti-OseIF3f antibody (GenScript). Total proteins were extracted from developing panicles with a grinding buffer (150 mM NaCl, 50 mM Tris-Cl, pH 7.5, 0.1% NP-40, 10 mM MgCl_2_, and 10 mM PMSF) and used to preform Western blotting as described (Deng and Wang, 2007).

### Cytological Analysis

For GUS histochemical staining, fresh florets from two independent *OseIF3f* promoter::GUS transgenic lines were stained with the staining buffer (Jefferson et al., 1987) at 37°C for at least 8 h, and then immersed in 95% ethanol until they became transparent. Photos were taken under the Leica S8APO StereoZoom Microscope (Leica Microsystems). The stained florets were embedded in Paraplast (Sigma), and sectioned on Leica RM2235 microtome as described (Song et al., 2007).

To estimate pollen viability, mature pollen grains and anthers from randomly-selected florets of each RNAi lines and control plants were stained in Alexander solution (Alexander, 1969). For cytological analysis of pollen development, immature (2.5–7 mm in length), and mature florets from WT and two RNAi lines (6L10 in T1 generation and 3L5 in R1 generation) were fixed in Carnoy’s solution for at least 4 h. Male meiotic spreads were prepared and stained with 4’,6-diamidino-2-phenylindole (DAPI) as described previously (Ross et al., 1996; Chen et al., 2005). Unicellular microspores and bicellular pollen were stained in 1% Aceto carmine solution (dissolved in 45% acetic acid). Mature pollen grains were stained by 1 μg/ml DAPI. Stained pollen grains were observed under the Axio Imager A1 microscope. Each test was repeated three times and the total number of counted pollen grains was about 500–2000 in each case.

## Results

### OseIF3f Is a Subunit of eIF3 in Rice

Studies of the mammalian eIF3 complex have demonstrated that eIF3f is one of the six subunits necessary for formation of a functional and stable eIF3 complex and eIF3f regulates protein synthesis in a cell-type-specific manner. (Hinnebusch, 2006; Sanchez et al., 2013). Rice eIF3f shows a higher expression level in developing pollen than that in mature pollen or germinated pollen (Wei et al., 2010). Therefore, we wondered whether eIF3f might be important for microgametogenesis in rice.

We first analyzed the sequences of eIF3f in rice. According to the gene annotation from the Rice Genome Annotation Project Database (RGAP)^1^, rice eIF3f is encoded by a single-copy gene (RGAP Locus: Os05g01450), which consists of 6 exons and 5 introns (**Supplementary Figure S1A**). The protein encoded by the Os05g01450 gene consists of 284 amino acids and contains the conserved Mpr1p and Pad1p N-terminal (MPN) domain (**Supplementary Figure S1B**).

**FIGURE 1:**
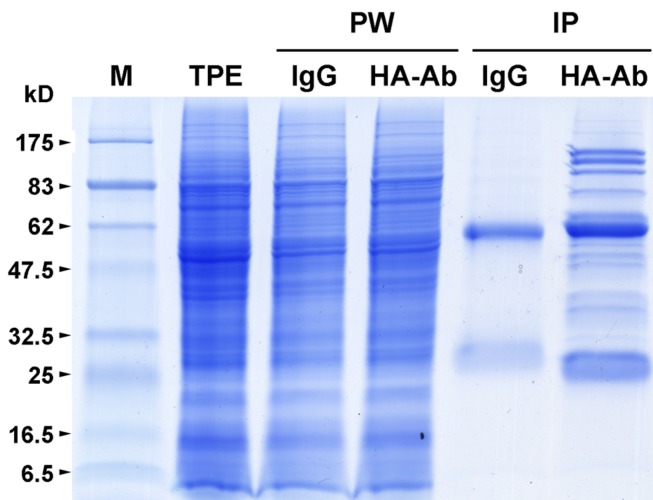
**Immunoprecipitation of HA-tagged OseIF3f from panicle protein extracts.** Total protein extracts (TPE) prepared from developing panicles of transgenic rice plants that overexpress the hemagglutinin (HA)-tagged rice eukaryotic translation initiation factor 3 subunit f (OseIF3f) were immunoprecipitated with mouse IgG coupled to Dynabeads Protein A (IgG) or a monoclonal anti-HA antibody coupled to Dynabeads Protein A (HA-Ab). TPE, unbound proteins washed away with the cold lysis buffer (PW) and Immunoprecipitates (IP) of HA-OseIF3f and IgG were separated by SDS-PAGE and visualized by Coomassie brilliant blue staining. Molecular weight standards (M) are marked on the left edge of the SDS-PAGE gels; kD, kiloDalton.

To confirm whether Os05g01450 encodes a subunit of the rice eIF3 complex, we carried out three independent replicates of immunoprecipitation to identify proteins that interact with its encoded protein (OseIF3f) by using anti-HA antibodies and total protein extracts prepared from developing panicles of transgenic rice lines that overexpress the HA-tagged ORF of Os05g01450 (**Supplementary Figure S2**). Excluding the protein bands present in the immunoprecipitates of IgG (the negative control), a number of protein bands with different molecular weight were shown to co-immunoprecipitate with HA-OseIF3f in panicles (**Figure 1**). Protein identification using mass spectrometry showed that almost all of the OseIF3f-interacting proteins present in all three replicates of HA-OseIF3f immunoprecipitation but absent from IgG immunoprecipitates were eIF3 subunits, including eIF3a, eIF3b, eIF3c, eIF3d, eIF3e, eIF3f, eIF3g, eIF3h, eIF3i, eIF3k, eIF3l, and eIF3m (**Supplemental Table S2**). This result indicates that OseIF3f is a subunit of the eIF3 complex, which consists of at least 12 subunits in rice.

### *OseIF3f* Is Highly Abundant in Developing Anthers

**FIGURE 2:**
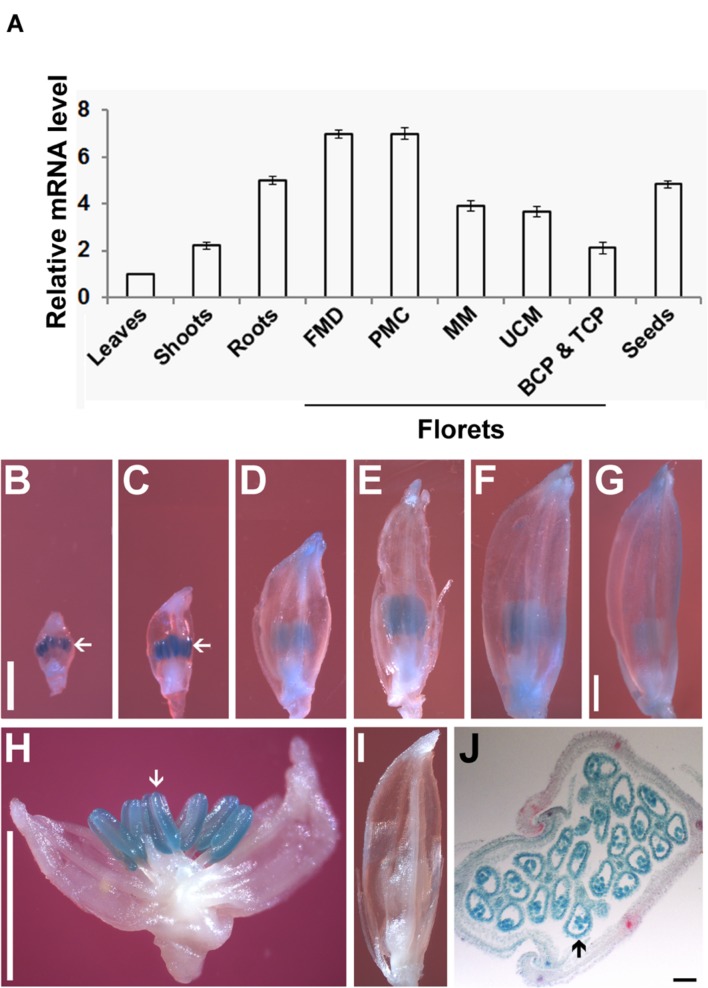
**Expression pattern of *OseIF3f.*** **(A)** Real-time qRT-PCR analysis of *OseIF3f* mRNA levels in leaves (from 30-day-old seedlings), roots (from 3-day-old seedlings), shoots (from 3-day-old seedlings), developing seeds (2–6 days after flowering), and florets at the floral bud morphological differentiation stage (FMD, florets of <0.9 mm in length from panicles of <15 mm in length), the pollen mother cell formation stage (PMC, florets of 0.9–2.5 mm from panicles of 15–50 mm), male meiotic stage (MM, florets of 2.5–5 mm in panicles of 50–100 mm), the unicellular microspore stage (UCM, florets of 5–7 mm in panicles of > 100 mm), the bicellular and tricellular pollen stage (BCP and TCP, florets of 7 mm). Three biological samples were used for qRT-PCR analyses of individual organs. Leaf samples were used as calibrator to normalize the mRNA levels in different organs. **(B–J)** GUS staining of developing florets from WT plants **(I)** and transgenic lines **(B–H,J)** transformed with the OseIF3f promoter::GUS construct, including florets at PMC **(B)**, early **(C,H,I)**, middle **(D)**, and late **(E)** stages of meiosis, UCM **(F)** and BCP and TCP **(G)** and the cross section of a developing floret **(J)**. Arrows indicate floret organs with intense GUS staining. Scale bars = 1 mm in **(B)** for **(B–F,I)**, in **(G)** for **(G)**, and in **(H)** for **(H)**. Bar = 50 μm in **(J)**.

To analyze the expression pattern of *OseIF3f*, we performed real-time qRT-PCR using total RNAs extracted from various organs of rice plants. *OseIF3f* mRNAs were detected in all of the organs examined (**Figure 2A**). The highest level of expression was found in florets at the floral bud morphological differentiation stage and those at the pollen mother cell formation stage (**Figure 2A**). *OseIF3f* showed high expression also in roots and developing seeds, with reduced expression in florets at the male meiotic stage and at the unicellular microspore stage (**Figure 2A**). *OseIF3f* was relatively weakly expressed in florets at the bicellular and tricellular pollen stages, shoots, and leaves (**Figure 2A**). Temporal and spatial expression analysis using *OseIF3f* promoter::GUS transgenic plants further revealed that *OseIF3f* had the highest abundance in developing anthers at the pollen mother cell formation stage and the early stage of meiosis (**Figures 2B–I**). Strong GUS staining was detected in pollen as well as in anther wall layers (**Figure 2J**). In contrast, GUS staining was very weak or undetectable in leaves, roots and shoots (data not shown). The reason *OseIF3f* promoter::GUS activity only partially reflected the mRNA expression pattern of *OseIF3f* may be that regulatory elements necessary for expression in vegetative tissues were located in regions other than the 2-kb genomic DNA sequence upstream of ORF (the 2-kb region was defined as *OseIF3f* promoter in our construct). High-level expression of *OseIF3f* in developing anthers suggests this gene might play an important role in male reproductive development.

### OseIF3f Is Localized to Endoplasmic Reticulum and Cytosol

**FIGURE 3:**
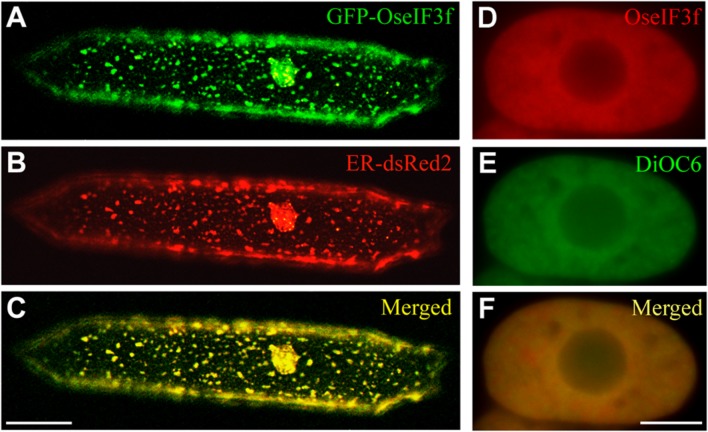
**Subcellular localization of OseIF3f.** **(A–C)** Transient expression of the GFP-OseIF3f fusion protein and the ER-resident red fluorescent protein (ER-dsRed2) in an onion epidermal cell. Scale bar = 50 μm. **(D–F)** Immunofluorescence localization of HA-OseIF3f in a rice root cortical cell stained with the ER-specific dye DiOC6. Scale bar = 10 μm.

For understanding the role of OseIF3f in rice, we also performed subcellular localization analysis. Transient expression of the GFP-OseIF3f fusion construct in onion epidermal cells revealed that the green signals from the GFP-OseIF3f proteins displayed a network-like or punctate distribution throughout the cytosol (**Figure 3A**), analogous to the localization pattern of the Endoplasmic Reticulum (ER). Cotransformation with a construct expressing an ER-resident red fluorescent protein (ER-dsRed2; Zhang et al., 2005) showed that the distribution of GFP-OseIF3f was partially overlapped with the red signals of the ER (**Figures 3A–C**), indicating that OseIF3f was localized to the endoplasmic reticulum as well as the cytosol. Furthermore, we immunolocalized OseIF3f with the anti-HA antibody and rice root cells from the transgenic lines overexpressing the HA-tagged OseIF3f protein. The hybridization signals of HA-OseIF3f (red) displayed partial overlap with the staining signals of the ER-specific dye DiOC6 (green) (**Figures 3D–F**), confirming the localization of OseIF3f to the ER and the cytosol in rice cells.

### Knockdown of *OseIF3f* Causes Pollen Abortion

We used double-stranded RNAi approach to address the *in vivo* function of *OseIF3f*. To ensure the efficacy and specificity of RNAi, two RNAi vectors, namely p3OseIF3fi and p6OseIF3fi, were constructed, respectively, using the 125–668 bp and 566–855 bp ORF fragments of *OseIF3f*, which share almost no similarity to other sequences in rice cDNA database. After *Agrobacterium*-mediated transformation of rice and confirmation of transgene integration into the rice genome, five transgenic RNAi lines, namely 6L2, 6L10, 6L11, 6L17 (obtained by transformation with p6OseIF3fi), and 3L5 (from p3OseIF3fi), and one transgenic negative control (TNC) line were randomly chosen for further analysis.

**FIGURE 4:**
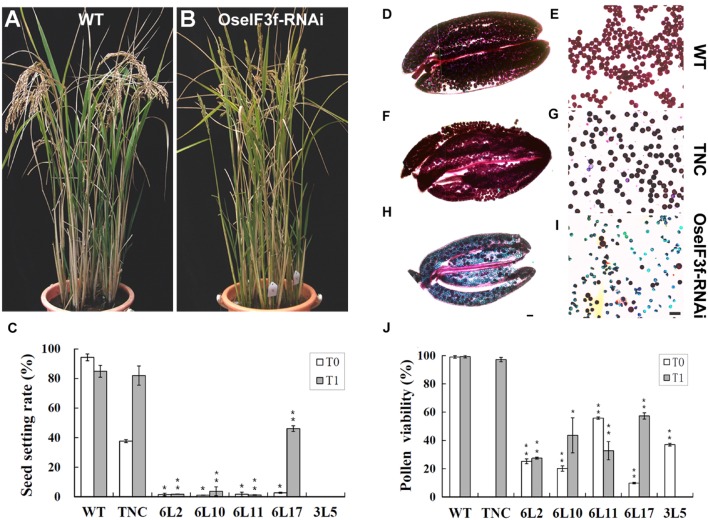
**Seed production and pollen viability of OseIF3f-RNAi lines compared with WT and transgenic negative control (TNC) plants.** **(A,B)** Plant morphology of WT **(A)** and OseIF3f-RNAi (6L10 in T1 generation) plants **(B)** at the mature stage. Panicles of WT bend down, while panicles of OseIF3f-RNAi remain upright because of fertility decline. **(C)** Mean seed setting rates in T0 and T1 generation. The TNC plants in the T0 generation showed reduced seed production, which was a negative effect of rice transformation and tissue culture. This effect disappeared in rice T1 plants. **(D–I)** Alexander staining of anthers **(D,F,H)** and pollen grains **(E,G,I)** from WT plants **(D,E)**, TNC plants in T1 generation **(F,G)**, and the 6L10 OseIF3f-RNAi plants in T1 generation **(H,I)**. Scale bars = 50 μm, in **(H)** for **(D,F,H)**, in **(I)** for **(E,G,I)**. **(J)** Statistical results of pollen viability for OseIF3f-RNAi lines compared with WT or TNC plants. Student’s *t*-test was used to compare the difference between the wild-type and each OseIF3f-RNAi line. “^∗^” and “^∗∗^” indicates *P* values generated by student’s *t*-test <0.05 and <0.01, respectively.

Compared to WT and TNC plants, all OseIF3f-RNAi lines showed no apparent abnormalities during vegetative growth, but their seed setting rates were greatly decreased in both the T0 and T1 generation (**Figures 4A–C**; **Supplementary Figure S3A**). The RNAi line 3L5 was fully sterile and four other lines (6L2, 6L10, 6L11, and 6L17) showed partial sterility (**Figure 4C**). Given that OseIF3f was highly expressed in developing anthers, we, therefore, wondered whether the reduction in fertility involved pollen abortion in the OseIF3f-RNAi lines. We compared the viability of mature pollen grains among WT, the TNC line and the RNAi lines by Alexander staining (Alexander, 1969). Anthers from the WT and TNC plants were filled by viable pollen grains, which were stained red in Alexander solution (**Figures 4D–G,J**). In comparison, a higher percentage of pollen grains from the RNAi anthers was blue-colored, shrunken, and variable in size and shape, indicating that pollen viability was significantly decreased in the RNAi lines (**Figures 4H–J**; **Supplementary Figure S3B**).

**FIGURE 5:**
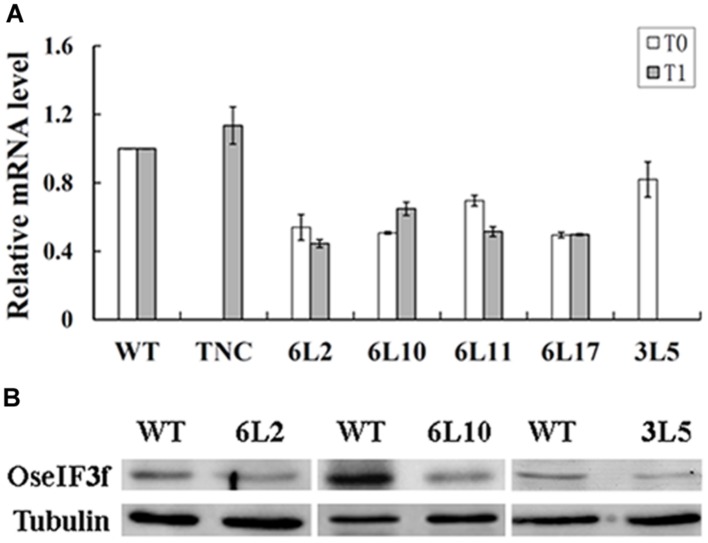
**Expression levels of OseIF3f in OseIF3f-RNAi lines compared with those in WT or TNC plants.** **(A)** Relative levels of *OseIF3f* mRNAs in T0 and T1 generation detected by real-time qRT-PCR. Real-time qRT-PCR amplification was performed in triplicate. WT samples were used as calibrator to normalize the mRNA levels in different RNAi lines. **(B)** Western blotting analysis of OseIF3f protein levels in 6L2, 6L10 (T1 generation), and 3L5 (T0 generation) compared with WT using the anti-OseIF3f antibody (**Supplementary Figure S2**).

To investigate whether pollen abortion was related to knockdown of *OseIF3f* expression in these RNAi lines, we used total RNAs extracted from developing panicles to perform real-time qRT-PCR to evaluate the endogenous level of *OseIF3f* transcripts. The transcript levels of *OseIF3f* in the TNC line were similar to those in WT (**Figure 5A**). In contrast, the *OseIF3f* transcripts in all the five RNAi lines were obviously down-regulated (**Figure 5A**). The Pearson’s r test showed strong correlation between pollen abortion and the decrease in *OseIF3f* mRNA levels (*r* = 0.93 for the T1 generation). In addition, Western blotting revealed that the levels of the OseIF3f protein in developing panicles of the RNAi lines were also lower than those of WT (**Figure 5B**). These results confirmed that RNAi-mediated knockdown of *OseIF3f* led to the reduction in pollen viability, which may partly contributed to the decrease in seed production.

### OseIF3f Is Required for Microgametogenesis

**FIGURE 6:**
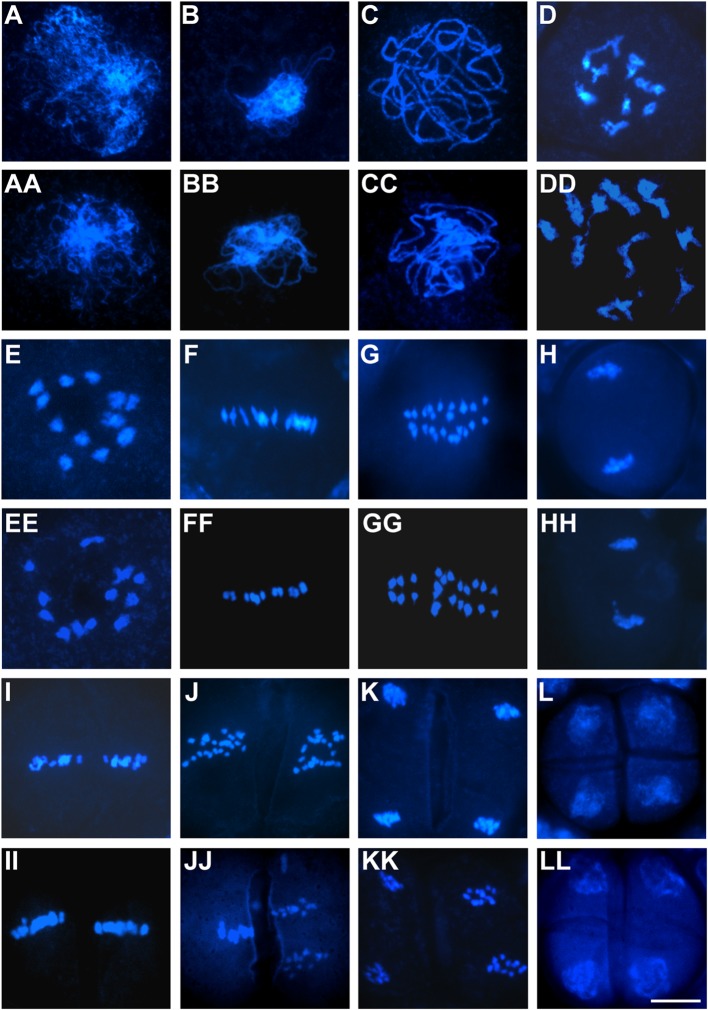
**Male meiosis in OseIF3f-RNAi lines compared with WT.** Meiotic chromosome spreads were prepared from WT **(A*–*L)** and OseIF3f-RNAi **(AA–LL)** male meiocytes and then stained with DAPI. Stages include leptotene **(A,AA)**, zygotene **(B,BB)**, pachytene **(C,CC)**, diplotene **(D,DD)**, diakinesis **(E,EE)**, metaphase I **(F,FF)**, anaphase I **(G,GG)**, telophase I **(H,HH)**, metaphase II **(I,II)**, anaphase II **(J,JJ)**, telophase II **(K,KK)**, and tetrad stage **(L,LL)**. Scale bar = 10 μm in **(LL)** for **(A–LL)**.

To evaluate whether aborted pollen grains resulted from defects in microsporogenesis or microgametogenesis, we carried out detailed cytological analysis of pollen development using the two representative OseIF3f-RNAi lines (6L10 and 3L5) compared with the WT control. Observations of male meiosis by DAPI-staining of chromosome spreads showed that similar to the process occurring in WT pollen mother cells (**Figures 6A–L**), homologous chromosomes synapsed, condensed and formed 12 bivalents in prophase I in male meiocytes from the two RNAi lines (**Figures 6AA–EE**). These bivalents aligned on the metaphase plate in metaphase I (**Figure 6FF**). Homologous chromosomes in each bivalent separated in anaphase I (**Figure 6GG**) and reached the opposite poles in telophase I (**Figure 6HH**). Afterwards, the RNAi meiocytes underwent the second meiotic division normally (**Figures 6II–KK**), which led to the formation of tetrads with four daughter cells of equal size (**Figure 6LL**). These observations demonstrated that meiotic chromosome behavior seemed not disrupted in the OseIF3f-RNAi lines, indicating that OseIF3f may not be necessary for microsporogenesis.

**FIGURE 7:**
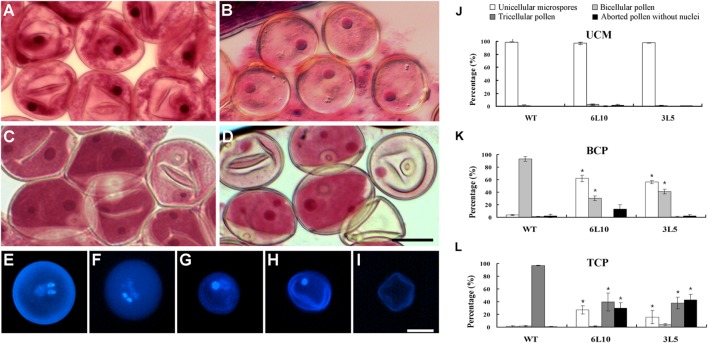
**Microgametogenesis in OseIF3f-RNAi lines compared with WT.** **(A–D)** Unicellular microspores **(A,B)** and bicellular pollen **(C,D)** in WT **(A,C)** and OseIF3f-RNAi **(B,D)** anthers stained with Aceto carmine. **(E–I)** DAPI-stained pollen from mature anthers of WT **(E)** and OseIF3f-RNAi **(F–I)**, including normal tricellular pollen **(E,F)**, arrested bicellular pollen **(G)**, arrested unicellular pollen **(H)**, and aborted pollen without nuclei **(I)**. Scale bars = 20 μm in **(D)** for **(A–D)**, in **(I)** for **(E–I)**. **(J–L)** Percentages of unicellular microspores, bicellular, tricellular, and aborted pollen in WT, 6L10 and 3L5 anthers at UCM, BCP, and TCP. Student’s *t*-test was used to compare the difference between the wild-type and each OseIF3f-RNAi line. “^∗^” indicates *P* values generated by student’s *t*-test <0.05.

We next compared the process of microgametogenesis between WT and the two RNAi lines. In rice, pollen grains enclosed in the same anther usually develop synchronously; thus immature pollen grains at different developmental stages can be collected separately, followed by staining with Aceto carmine. The two RNAi lines showed no obvious defects when unicellular microspores were formed and enlarged after meiosis (**Figures 7A,B**). The percentage of normal unicellular microspores in 6L10 (97%, *n* = 1465) and 3L5 (98%, *n* = 2190) were similar to that in WT (98%, *n* = 865) (**Figure 7J**). As unicellular microspores underwent the first round of pollen mitosis to produce bicellular pollen containing the large vegetative cell with a dispersed nucleus and the small generative cell with a condensed nucleus, significant differences were detected between WT and the RNAi lines (**Figures 7C,D**). At least 90% of WT pollen had two nuclei, namely the large nucleus from the vegetative cell and the small nucleus from the generative cell (**Figure 7K**). However, in 6L10 and 3L5, 58% and 56% of pollen contained only one nucleus (**Figure 7K**), respectively, suggesting these pollen grains may be arrested at the unicellular stage.

We further used DAPI staining to examine pollen grains of mature florets, which were collected just before anthesis. WT mature florets contained a high proportion (more than 90%, *n* = 1252) of tricellular pollen, which had one large diffuse-staining nucleus of the vegetative cell and two condensed nuclei of the two sperm cells generated by the second round of pollen mitosis (**Figure 7E**). In contrast, only 36% and 38% of mature pollen grains in 6L10 (*n* = 1014) and 3L5 (*n* = 1406), respectively, were tricellular (**Figures 7F,L**). The two RNAi lines showed a great increase in the percentage of aberrant pollen (**Figure 7L**), including arrested bicellular pollen (**Figure 7G**), arrested unicellular pollen (**Figure 7H**), and aborted pollen without nuclei (**Figure 7I**). These results demonstrated knockdown of *OseIF3f* resulted in abnormalities in post-meiotic pollen formation, which was related to pollen abortion in the OseIF3f-RNAi lines. Therefore, *OseIF3f* may play a role in microgametogenesis.

## Discussion

### Plant eIF3 Complex May Have a Subunit Composition Identical to that of Human

As the largest and most complex initiation factor for eukaryotic protein synthesis, the eIF3 complex has been isolated and characterized in many eukaryotes, such as *Saccharomyces cerevisiae*, *Homo sapiens*, *Triticum aestivum*, and *Arabidopsis thaliana* (Hinnebusch, 2006; Marchione et al., 2013). Human eIF3 comprises the maximum number of subunits (13 different subunits), including eIF3a to eIF3m. The eIF3 complex of budding yeast shares six subunits (eIF3a, b, c, g, i, and j) with human eIF3, but also has three unique subunits without identifiable homologues in human. The functional core of yeast eIF3 is composed of eIF3a, b, c, g, and i, which are crucial for translation initiation *in vivo*. However, in human, the former three conserved subunits (eIF3a, b, and c) and three non-conserved subunits (eIFe, f and h) are vital components for formation of the active core of eIF3. Wheat and *Arabidopsis* eIF3 consists of eIF3a-i, k and l, which is more similar to the human eIF3 complex than to the yeast one (Burks et al., 2001). In this study, we identified twelve eIF3 subunits from immunoprecipitates of OseIF3f, including eIF3a–i, k, l, and m. This result shows that the subunit composition of rice eIF3 closely resembles that of Wheat and *Arabidopsis* eIF3, indicating that eIF3 components are highly conserved among plants. OseIF3f immunoprecipitates contained eIF3m, which was not detected in the purified *Arabidopsis* eIF3 complex. However, genes encoding the eIF3m subunit are existed in both the *Arabidopsis* and rice genomes. There are two *Arabidopsis* genes for eIF3m, namely At3g02200 and At5g15610, which encode proteins share 56% identity with the rice eIF3m protein (Os04g01290). In addition, eIF3j seems to be included in neither the *Arabidopsis* nor the rice eIF3 complex compared to human eIF3, although two genes have been annotated to encode eIF3j in both *Arabidopsis* and rice^2^. The two rice eIF3j proteins (Os02g02990 and Os05g41630) share 56% identity with the two *Arabidopsis* eIF3j proteins (At1g66070 and At5g37475) but show only 35% identity to human eIF3j. Recently, study of human eIF3 by mass spectrometry reveals that eIF3m and eIF3j are located on the periphery of the eIF3 complex and are loosely associated with other subunits (Damoc et al., 2007; Zhou et al., 2008). Given that the encoding genes of the two subunits are present in the rice and *Arabidopsis* genomes, it is possible that we only obtained incomplete eIF3 complex containing stable subunits but losing some of the labile subunits. The intact eIF3 complex in rice, as well as in other plants, may also consist of all the 13 non-identical subunits of human eIF3.

### Role of OseIF3f in Rice

Molecular genetic studies of *Arabidopsis* eIF3 subunits have shown that mutation in eIF3e, eIF3f, or eIF3h does not affect pollen formation or maturation, but leads to defects in pollen germination and/or tube growth and consequently causes reduction in male gametophytic transmission (Xia et al., 2010; Roy et al., 2011). These results indicate that the three *Arabidopsis* eIF3 subunits serve essential functions in pollen germination and tube growth. In this study, we reveal that the rice eIF3 subunit f has a role in post-meiotic pollen formation. Although it is uncertain whether OseIF3f is also necessary for pollen germination, OseIF3f might be more important for microgametogenesis than for pollen germination because OseIF3f was expressed at least three-fold higher levels in unicellular microspores and bicellular pollen than in mature tricellular pollen or germinated pollen (Wei et al., 2010). Interestingly, other subunits of the rice eIF3 complex, including eIF3a–e, g–i, l, and m, show a similar expression pattern during post-meiotic pollen development and germination (Wei et al., 2010), which suggests these subunits of rice eIF3 may also be involved in microgametogenesis.

The eIF3 complex plays a scaffolding role in translation initiation, thus regulating protein synthesis (Dong and Zhang, 2006; Hinnebusch, 2006). In mammals, eIF3f have been shown to serve as a regulator for translation during cell growth and apoptosis (Marchione et al., 2013). OseIF3f might possibly function in protein synthesis and regulation because OseIF3f was localized to the endoplasmic reticulum and the cytosol, which are the places for protein production, folding, and quality control. It will be interesting to determine whether OseIF3f contributes to microgametogenesis through regulating the translation of proteins required for pollen formation.

Considering that protein synthesis and regulation occurs during many cellular processes such as cell division and enlargement, it is reasonable to suppose that OseIF3f, a subunit of rice translation initiation factor 3, should function in various developmental processes in addition to microgametogenesis. Consistent with this assumption, *OseIF3f* mRNAs were detected in various organs including leaves, roots, shoots and panicles; however, *OseIF3f* showed the highest level of mRNA expression in immature florets and the strongest promoter::GUS activity in developing anthers. This expression pattern was similar to *OsGEN-L* and *OsRAD21-3*, the two identified genes involved in rice microgametogenesis. Furthermore, analogous to the situation for *OsGEN-L* or *OsRAD21-3*, knockdown of *OseIF3f* have no obvious effect on vegetative growth. Given that eIF3f does not directly participate in translation initiation (Masutani et al., 2007), it is possible that O*seIF3f* is not indispensable for vegetative organ development. Alternatively, lower expression levels of OseIF3f are necessary for vegetative growth than those for pollen development; thus the moderate decrease of OseIF3f expression in RNAi lines is inadequate for disrupting vegetative development. Nevertheless, *OseIF3f* may be essential for female reproductive development or/and seed development because (i) the OseIF3f-RNAi lines displayed a more dramatic reduction in seed setting rates than in pollen viability (**Figures 4C,J**) and (ii) *OseIF3f* showed a relatively high expression in developing seeds (**Figure 2A**). In addition, it is also possible that *OseIF3f* have a role in the development of anther wall layers since *OseIF3f* promoter::GUS staining was strong in these tissues and anther wall layers has been shown to be important for pollen fertility (Zhang et al., 2011). Further cellular and genetic studies are necessary for a better understanding of the function of OseIF3f in rice.

## Author Contributions

TW and ZD designed the research. QL and CG performed the experiments. QL, ZD, and CG analyzed the data. ZD, QL, and CG wrote the manuscript text.

## Conflict of Interest Statement

The authors declare that the research was conducted in the absence of any commercial or financial relationships that could be construed as a potential conflict of interest.

## Footnotes

1 http://rice.plantbiology.msu.edu/

2 http://www.uniprot.org/

## Supplementary Material

**FIGURE S1 | The exon–intron gene structure **(A)** and the protein domain structure **(B)** of Os05g01450.**

**FIGURE S2 | Western hybridization using the anti-OseIF3f antibody.** **(A)** Antibody validation by Western blotting. Western blotting was performed with total protein extracts isolated from developing panicles of wild-type (WT) rice. The produced anti-OseIF3f antibody (Ab) recognized a protein band between 25 and 35 kDa, corresponding to the size range of the OseIF3f protein. However, this protein band was absent in the Western blot probed with the preimmune rabbit serum (P), indicating that this band was the OseIF3f protein. **(B)** Confirmation of HA-OseIF3f expression in OseIF3f-overexpressed transgenic lines. The anti-OseIF3f antibody was used to probe total protein extracts from developing panicles of WT and OseIF3f-overexpressed transgenic lines (OE). The protein band of OseIF3f was present both in WT and OE extracts, while an additional protein band with a molecular weight greater than that of OseIF3f was detected only in OE extracts. This higher molecular weight protein was the hemagglutinin (HA)-tagged OseIF3f protein (HA-OseIF3f). M, Fermentas unstained protein molecular weight marker (Thermo Fisher Scientific); kD, kiloDalton.

**FIGURE S3 | The statistical results of seed setting rates **(A)** and pollen viability **(B)** for all OseIF3f-RNAi lines we analyzed.**
