# Supplementary figures and images for: The Rice Eukaryotic Translation Initiation Factor 3 Subunit f (OseIF3f) Is Involved in Microgametogenesis
Source: Front Plant Sci. 2016 Apr 26;7:532. doi: 10.3389/fpls.2016.00532 (PMC4844609; doi:10.3389/fpls.2016.00532)

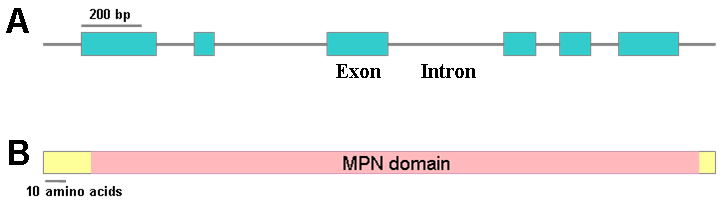

Supplement: Supplementary file 1 [file Image_1.TIF]

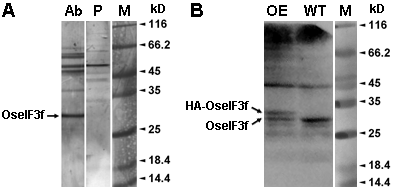

Supplement: Supplementary file 2 [file Image_2.TIF]

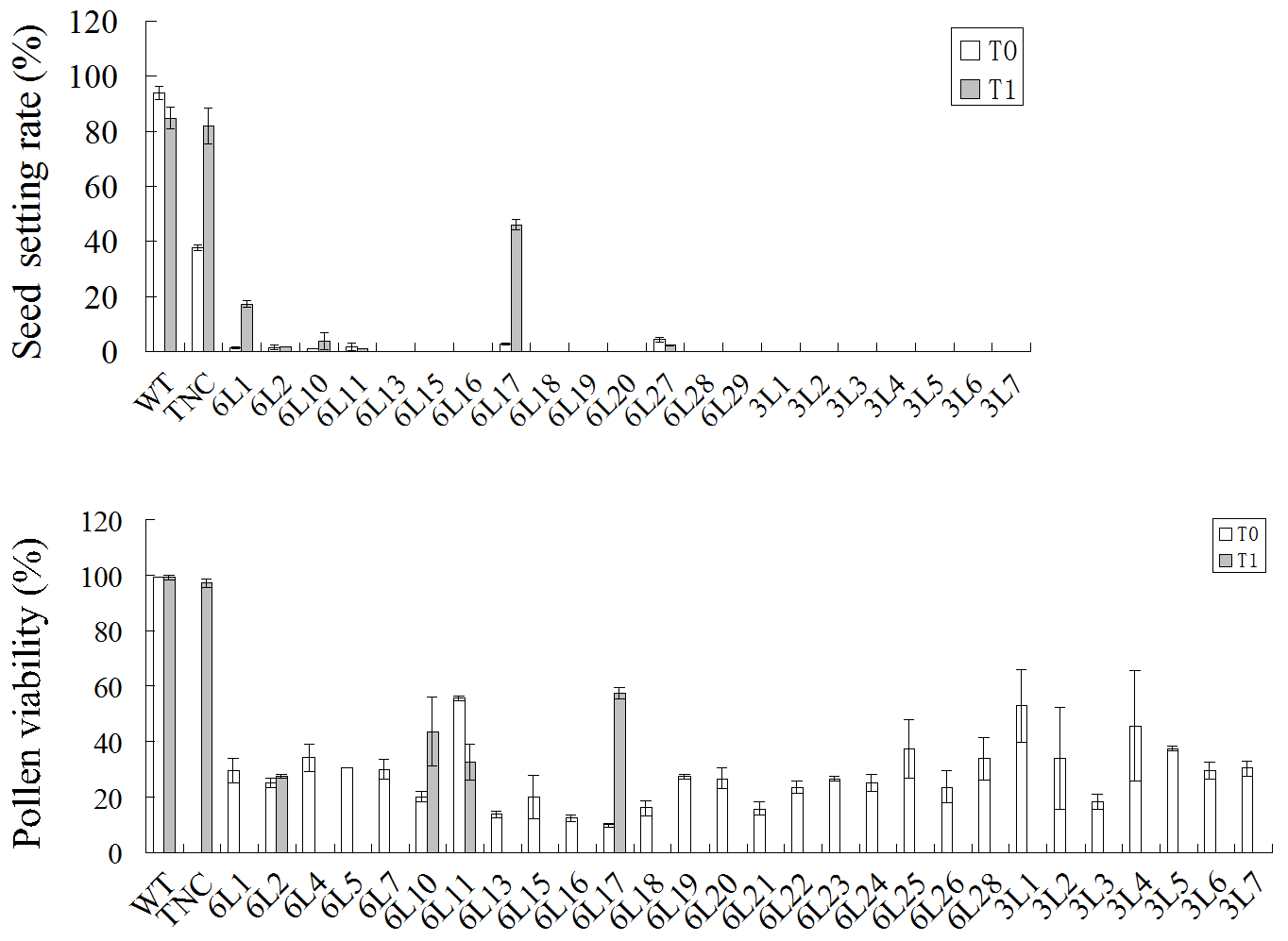

Supplement: Supplementary file 3 [file Image_3.TIF]
