# Supplementary material for: The Rice Eukaryotic Translation Initiation Factor 3 Subunit f (OseIF3f) Is Involved in Microgametogenesis
Source: Front Plant Sci. 2016 Apr 26;7:532. doi: 10.3389/fpls.2016.00532 (PMC4844609; doi:10.3389/fpls.2016.00532)
Supplement: Supplementary file 4 [file Table_1.DOCX]

**Supplemental Table 1. A list of primers used in this study**

| **Primer Name** | **Sequence (5'-3')** | **Restriction Enzyme** | **Usage** |
| --- | --- | --- | --- |
| P1F | AAAGGATCCACCATG*TACCCATACGACGTTCCAGACTACGCT*GCTGCGGCGGAGGGCCCC | *BamH*I | pHA-OseIF3f |
| P1R | ACGAGCTCAGCAGTCGTGCGAATGAA | *Sac*I | pHA-OseIF3f |
| P2F | AAACTGCAGAATGTGCTCAGTCACTGCAA | *Pst*I | pOseIF3f::GUS |
| P2R | AAAAGATCTGAGGTAGAGGGGAAGAGGAG | *Bgl*II | pOseIF3f::GUS |
| P3F | TAGGTACCCCAATGATCTGGAAGGAATG | *Kpn*I | p6OseIF3fi |
| P3R | TAGGATCCAGCAGTCGTGCGAATGAA | *BamH*I | p6OseIF3fi |
| P4F | TATGGCGCGCCCCAATGATCTGGAAGGAATG | *Asc*I | p6OseIF3fi |
| P4R | ACGAGCTCAGC AGTCGTGCGAATGAA | *Sac*I | p6OseIF3fi |
| P5F | CTCGGTACCACTAGTCGTCATCGGCACCCTCCT | *Kpn*I, *Spe*I | P3OseIF3fi |
| P5R | ATCGGATCCGAGCTCGGTGCTACACGCCCCTCC | *BamH*I, *Sac*I | P3OseIF3fi |
| P6F | GAAAAGTTGGATTTGACATCCTGAA |  | OseIF3f qRT-PCR |
| P6R | GCCCCTCCACGACATCATC |  | OseIF3f qRT-PCR |
| P7F | CGTGTAGCACCGGACAATAAA |  | OseIF3f qRT-PCR |
| P7R | CACCAGTGCAAGATTATCCTGA |  | OseIF3f qRT-PCR |
| P8F | CGGCTACCACATCCAAGGAA |  | 18s rRNA qRT-PCR |
| P8R | TGTCACTACCTCCCCGTGTCA |  | 18s rRNA qRT-PCR |
| P9F | GCTGACCACACCTAGCTTTGG |  | Tubulin qRT-PCR |
| P9R | AGGGAACCTTAGGCAGCATGT |  | Tubulin qRT-PCR |
| P10F | ATTCTAGAA*GCTGCTGCTGCTGCTGCT*ATGGCTGCGGCGGAGGGC | *Xba*I | pGFP-OseIF3f |
| P10R | TAGGATCCAGCAGTCGTGCGAATGAA | *BamH*I | pGFP-OseIF3f |

The nucleotide sequences underlined are restriction enzyme cutting sites for each primer.

The nucleotide sequences in italics in P1F encode HA.

The nucleotide sequences in italics in P10F encode six alanines.
